# Pemphigus Foliaceus in an 11-Year-Old Mexican Girl with Response to Oral Dapsone

**DOI:** 10.1155/2013/291256

**Published:** 2013-12-12

**Authors:** Martha Elena García-Meléndez, Kristian Eichelmann, Julio César Salas-Alanís, Minerva Gomez-Flores, Jorge Ocampo-Candiani

**Affiliations:** Department of Dermatology, University Hospital “José Eleuterio González," Universidad Autónoma de Nuevo León (UANL), Avenida Francisco I. Madero Pte. s/n y Avenida Gonzalitos, Colonia Mitras Centro, 64460 Monterrey, NL, Mexico

## Abstract

Pemphigus foliaceus (PF) is rarely described in the pediatric population with less than 40 cases reported in the literature. We report the case of an 11-year-old girl who was diagnosed with PF after 6 months of starting with symptoms and who responded well to therapy with oral dapsone. Although therapeutic guidelines for PF in children are lacking, oral corticosteroids in combination with dapsone have proven to be effective as first-line treatment in this setting.

## 1. Introduction

Pemphigus foliaceus (PF) is an autoimmune, blistering skin disease, targeting desmoglein-1. There are two major categories: the endemic form is common in children in rural areas of Brazil where the bite of the black fly *Simulium nigrimanum* is thought to be the mode of transmission of the disease [1]. However, the sporadic form is rare in children, with less than 40 cases documented in the literature. Because of the rarity of the disease, evidence on the treatment and prognosis in children is lacking. We describe a girl with sporadic PF who responded well to dapsone.

## 2. Case Report

**Figure 1:**
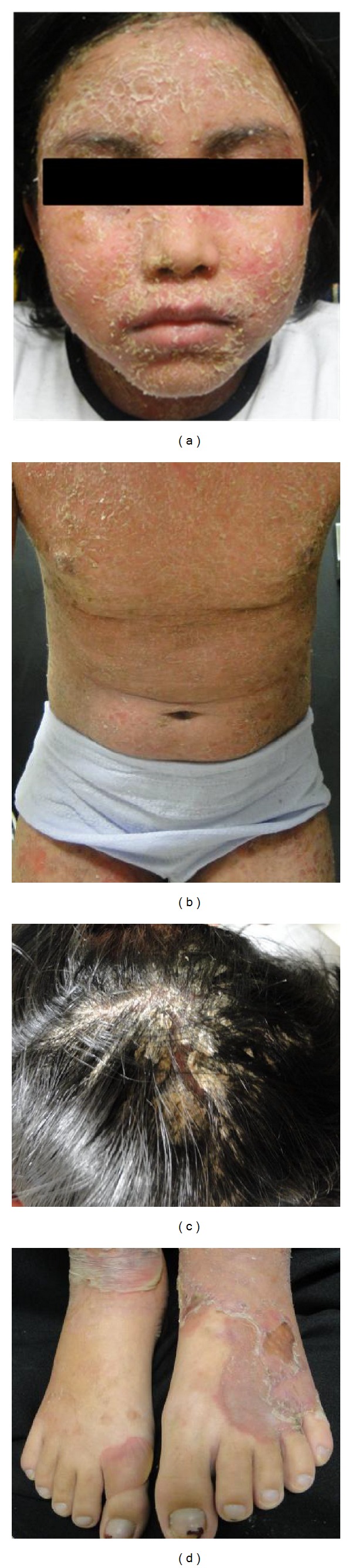
(a), (b), and (c) Patient showing scaling erythroderma with yellow crusts on the face, trunk, lower extremities, and scalp. (d) A single tense, nonhemorrhagic blister on the dorsal aspect of her right foot.

**Figure 2:**
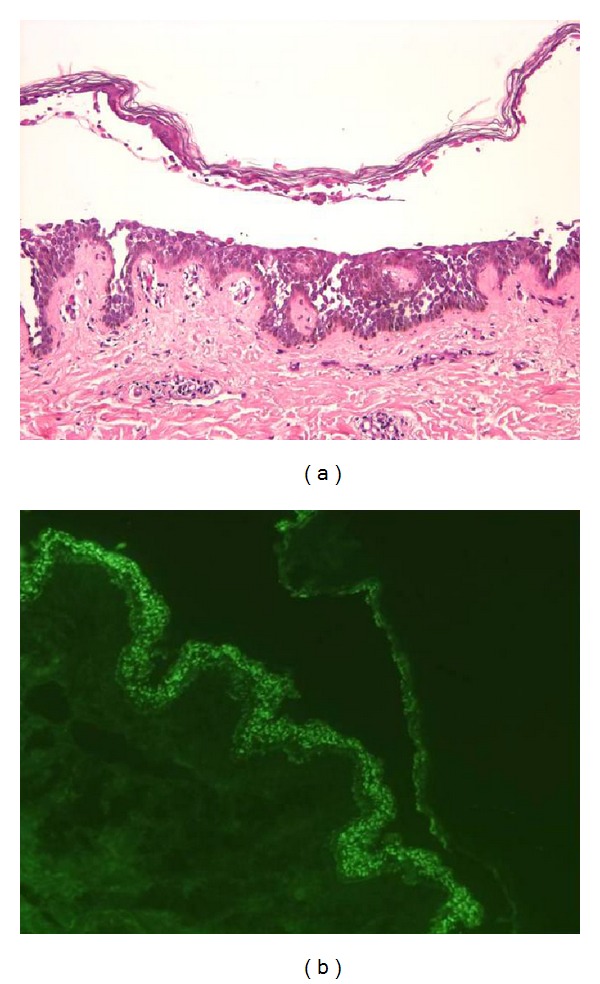
(a) Skin biopsy stained with H&E showing a subcorneal blister. (b) Direct Immunofluorescence demonstrating granular IgG deposits on epidermal keratinocytes showing a “beehive” pattern.

**Figure 3:**
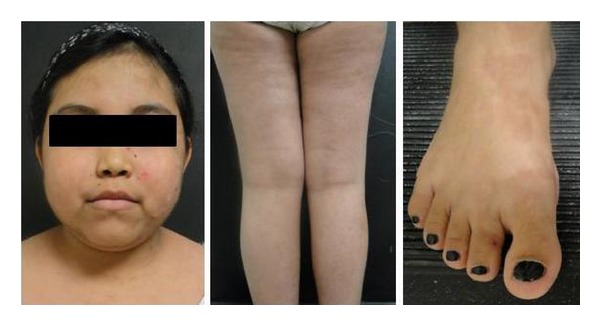
Patient showing complete remission of skin lesions after 9 months, with transitory cushingoid facies.

An 11-year-old Mexican girl presented with a six-month history of an asymptomatic generalized skin eruption involving the face, trunk, and extremities. The condition initially began as tense blisters on the face, which spontaneously resolved leaving erythema and scaling. She was treated with topical corticosteroids and systemic antibiotics without improvement. The lesions progressed slowly, eventually covering her entire skin surface, accompanied by general malaise, chills, and edema of the lower extremities. On physical examination, she had scaling erythroderma with yellow-greenish crusts involving her entire body surface (Figures 1(a)–1(c)). A single, tense blister was also noted on the dorsal aspect of her right foot (Figure 1(d)). The skin biopsy specimen showed a subcorneal bulla with upper epidermal acantholysis (Figure 2(a)). Direct immunofluorescence was positive for C3 and IgG deposits in the stratum spinosum with a beehive pattern (Figure 2(b)). Skin culture was positive for *Staphylococcus aureus*. Treatment was started with oral prednisone (1 mg/kg/day) and systemic dicloxacillin. Quantification of glucose-6-phosphate dehydrogenase levels was performed, and maintenance treatment with dapsone 50 mg/day was started. She responded satisfactorily over the following month and was continued on a regimen of dapsone 50 mg/day with tapered doses of prednisone. Our patient developed transitory Cushing's syndrome and remained free of disease after dapsone was discontinued, with a follow-up of nine months (Figure 3).

## 3. Discussion

Pemphigus foliaceus is a blistering skin disease that rarely occurs in the pediatric population, with the exception of the endemic form observed predominantly in Brazilian children, known as Fogo selvagem [2]. Because blisters are superficial within the epidermis and the blister roof is too thin to allow fluid accumulation, patients usually present with erythematosquamous plaques and superficial erosions that may resemble an exfoliative dermatitis. Differential diagnoses include severe or disseminated forms of impetigo, seborrheic dermatitis, psoriasis, and staphylococcal scalded skin syndrome. Diagnosis is based on clinical and histopathological findings, mainly the presence of a subcorneal blister with positive DIF for IgG and C3. The target antigen in pemphigus foliaceus is desmoglein-1, a 160 kDa constituent of desmosomes [3]. Since there are very few cases of pemphigus foliaceus in children reported in the literature, evidence-based treatment guidelines are lacking. Systemic corticosteroids are the treatment of choice (1-2 mg/kg/day), with dapsone being the most commonly used adjuvant agent (50–100 mg/day), allowing a decrease in the dose of corticosteroids required to control the disease. Dapsone is a sulfonamide antibiotic which blocks the synthesis of dihydrofolic acid. It is mostly used for its therapeutic efficacy in skin diseases with abnormal accumulation of neutrophils [4]. However, its mechanism of action in antibody-mediated diseases such as pemphigus foliaceus remains unclear [5]. Patients with PF show a relatively benign course when compared with children with pemphigus vulgaris [6]. Our patient showed complete remission with these two agents combined with a long-term follow-up of 20 months free of the disease.
